# RETRACTED: Kanan et al. Empowering Low- and Middle-Income Countries to Combat AMR by Minimal Use of Antibiotics: A Way Forward. *Antibiotics* 2023, *12*, 1504

**DOI:** 10.3390/antibiotics14010084

**Published:** 2025-01-14

**Authors:** Mohammed Kanan, Maali Ramadan, Hanan Haif, Bashayr Abdullah, Jawaher Mubarak, Waad Ahmad, Shahad Mari, Samaher Hassan, Rawan Eid, Mohammed Hasan, Mohammed Qahl, Atheer Assiri, Munirah Sultan, Faisal Alrumaih, Areej Alenzi

**Affiliations:** 1 Department of Clinical Pharmacy, King Fahad Medical City, Riyadh 12211, Saudi Arabia; 2 Department of Pharmacy, Maternity and Children Hospital in Rafha, Rafha 76312, Saudi Arabia; maraalshammari@moh.gov.sa (M.R.); halshammari19@moh.gov.sa (H.H.); balznezi@moh.gov.sa (B.A.); pharm.d.jawaher@gmail.com (J.M.); 3 Department of Pharmacy, King Khalid University, Abha 61421, Saudi Arabia; waadalqaedi_0197@hotmail.com (W.A.); shahd14121@gmail.com (S.M.); 4 Department of Clinical Pharmacy, Jazan College of Pharmacy, Jazan 82726, Saudi Arabia; 201600591@stu.jazanu.edu.sa; 5 Department of Pharmacy, Nahdi Company, Tabuk 47311, Saudi Arabia; alanazi.re@nahdi.sa; 6 Department of Pharmacy, Armed Forces Hospital Southern Region, Mushait 62562, Saudi Arabia; mhm1417@hotmail.com (M.H.); assiriatheer19@gmail.com (A.A.); 7 Department of Pharmacy, Najran Armed Forces Hospital, Najran 66256, Saudi Arabia; phy.eissa@nafh.med.sa; 8 Al-Adwani General Hospital, Taif 26523, Saudi Arabia; pharm.muneera77@hotmail.com; 9 Department of Pharmacy, Northern Border University, Rafha 76313, Saudi Arabia; faisal.alrumaih@hotmail.com; 10 Department of Infection Control and Public Health, Regional Laboratory in Northern Border Region, Arar 73211, Saudi Arabia; araalenzi@moh.gov.sa

The journal retracts the article “Empowering Low- and Middle-Income Countries to Combat AMR by Minimal Use of Antibiotics: A Way Forward” [1], cited above. 

Following publication, concerns were brought to the attention of the Editorial Office regarding an overlap between this article [1] and another manuscript [2] under consideration at another publisher at the same time, produced by a different authorship group. 

In accordance with our complaints procedure, an investigation was conducted by the Editorial Office and the Editorial Board. Following discussions with the authors, the Editorial Board was unable to verify the authenticity of the authorship list or confirm the origins of this article [1]. As a result, the Editorial Board and the authors have decided to retract this article [1] in accordance with MDPI’s retraction policy (https://www.mdpi.com/ethics#_bookmark30, accessed on 28 November 2024).

This retraction was approved by the Editor-in-Chief of *Antibiotics*. 

The authors have agreed to this retraction.
